# Impact of age, race, and medication use on efficacy endpoints in a randomized controlled trial of topical sildenafil cream for the treatment of female sexual arousal disorder

**DOI:** 10.1093/sexmed/qfae079

**Published:** 2024-11-19

**Authors:** Isabella Johnson, Andrea Ries Thurman, Katherine A Cornell, Clint Dart, Jessica Hatheway, David R Friend, Andrew Goldstein

**Affiliations:** Daré Bioscience, San Diego, CA, United States; Daré Bioscience, San Diego, CA, United States; Strategic Science & Technologies, LLC, Cambridge, MA, United States; Premier Research, Morrisville, NC, United States; Daré Bioscience, San Diego, CA, United States; Daré Bioscience, San Diego, CA, United States; Daré Bioscience, San Diego, CA, United States

**Keywords:** female sexual arousal disorder, fsad, sildenafil, hormonal contraception, age, race

## Abstract

**Background:**

A study of topical Sildenafil Cream 3.6% was completed among healthy premenopausal women with female sexual arousal disorder.

**Aims:**

To compare efficacy endpoints based on product use in pre-planned and post-hoc subsets of age, race, and medication use.

**Methods:**

Phase 2b, exploratory, randomized, placebo-controlled, double-blind study of Sildenafil Cream, 3.6% among healthy premenopausal women with female sexual arousal disorder (FSAD). Eligible participants were randomized 1:1 to Sildenafil versus Placebo Cream and used investigational product for 12 weeks.

**Outcomes:**

The co-primary efficacy endpoints were the change from baseline, at week 12, in the Arousal Sensation (AS) domain of the Sexual Function Questionnaire (SFQ28) and Question 14 (Q14) of the Female Sexual Distress Scale – Desire, Arousal, Orgasm (FSDS-DAO). The secondary efficacy endpoint was the change from baseline at week 12 in the mean number of satisfactory sexual events (SSEs) reported in a daily diary. Exploratory efficacy endpoints included the Desire and Orgasm domains of the SFQ28.

**Results:**

Age group (≥18 years and ≤ 45 years versus >45 years), race group (White versus non-White), and baseline use/non-use of hormonal contraception did not significantly affect the co-primary endpoints of the SFQ28 AS domain and FSDS-DAO Q14 (*P* values >0.11). Non-White Sildenafil Cream users had an increase in SSEs at week 12 (0.7 ± 0.63) while non-white Placebo Cream users reported a decrease (−1.5 ± 0.58) (*P* = .02). Daily psychiatric medication use among women assigned to either Placebo or Sildenafil Cream resulted in lower SFQ28 Desire domain scores compared to non-users of these medications. Women who used study product only in un-partnered events had a larger improvement in their SFQ28 Orgasm domain scores at week 12 (2.39 ± 0.95) with Sildenafil Cream use compared to Placebo (−0.19 ± 0.75) (*P* = .06). Non-White women represented a higher proportion of un-partnered women and women who used IP only during un-partnered sexual events compared to White women (*P* < .01).

**Clinical Implications:**

These pre-planned subset analyses will help refine target populations in future studies of Sildenafil Cream, 3.6% for the treatment of FSAD.

**Strengths and Limitations:**

Subset analyses focused on variables pertinent to future target populations. The current study population was primarily educated non-Hispanic White women.

**Conclusion:**

Age and hormonal contraceptive use did not impact the efficacy of topical Sildenafil Cream. Daily psychiatric medication use decreased sexual desire in active and placebo users.

## Introduction

Female sexual arousal disorder (FSAD) is a persistent or recurrent inability to attain, or to maintain until completion of the sexual activity, an adequate lubrication-swelling response of sexual excitement that causes marked distress or interpersonal difficulty.^1^^,^^2^ Further, the symptoms cannot be caused by a co-existing medical or psychiatric condition, problems within the relationship, or the effects of a medication or other drug substances.^1^^,^^2^ FSAD is physiologically analogous to male erectile dysfunction (ED), characterized by impaired blood flow to genital tissues.

To date, there are no US Food and Drug Administration (FDA) approved pharmacological treatments for FSAD. Oral sildenafil citrate (Viagra®) has been approved since 1998 for the treatment of ED. Multiple studies testing oral sildenafil citrate for the treatment of female sexual dysfunction (FSD) (including heterogenous FSD populations consisting of women with hypoactive sexual desire disorder, female orgasmic disorder, and FSAD have shown therapeutic benefit only in clearly defined sub-populations,^3-9^ but the side effect profile was poor. Therefore, topical Sildenafil Cream, 3.6% (Sildenafil Cream) is being developed for the treatment of FSAD in women. The hypothesis is that by delivering sildenafil topically, specifically targeting genital anatomy central to the vascular arousal response, there will be less systemic exposure, followed by less systemic side effects, resulting in a more favorable safety profile.

The primary objective of the completed study was to evaluate the efficacy of Sildenafil Cream among healthy premenopausal women with FSAD.^10^ Prior to the performance of the study, various pre-specified subset analyses were defined to test the hypothesis that various demographic characteristics (eg, age, race) and medication use (hormonal contraceptives) had an impact on the primary efficacy variables in this study. After completion of the study, post-hoc analyses were performed to test the hypothesis that behavioral factors (eg, partnered versus un-partnered sexual events) and daily psychiatric medication use (eg, selective serotonin reuptake inhibitors (SSRIs)) had an impact on study efficacy endpoints.

## Methods

### Clinical study

As previously reported,^10-12^ the clinical trial was conducted at 49 sites in the United States and was approved by the Advarra Institutional Review Board (Pro00049161) and registered at ClinicalTrials.gov (#NCT04948151). Healthy premenopausal women, aged 18 years or older, and their sexual partners, if applicable, were screened for the study. Volunteers provided written informed consent prior to the performance of any study related procedures. Women of child-bearing potential used effective birth control throughout the study (eg, hormonal contraception, intrauterine devices, contraceptive implants). Condoms could be used to prevent sexually transmitted infections. Except for women with anxiety, depression, and attention deficit hyperactivity disorder (ADHD), patients with an axis I DSM-IV-TR disorder (eg, schizophrenia, bipolar disorder) were excluded. Participants diagnosed with anxiety, depression, or ADHD who were taking medical treatment had to be on a stable dose for at least the past 6 months to be considered for study participation. Women who changed the dose of their psychiatric medications during the trial, even if going to a lower dose or discontinuing treatment, were discontinued from the study. Participants who had any history of anti-psychotic therapy use within the last year were excluded unless the anti-psychotic medications (eg, risperidone) were given for a diagnosis of anxiety and/or depression, and their dosage had been stable for at least 6 months.

The patient health questionnaire-8 (PHQ-8) and the generalized anxiety disorder-7 (GAD-7) surveys were administered at screening and monthly until the enrollment visit (visit 4, V4). Women had to have a total score of <10 on both the PHQ-8 and the GAD-7 to enroll in the study. Finally, women were excluded if they had experienced a recent major life stress (e.g., loss of income, death of a family member) or had un-controlled hypertension, a history of serious cardiac events (eg, myocardial infarction, stroke), symptomatic orthostatic hypotension, or other serious medical co-morbidities.

Approximately 1 year into the study, we concluded that a major barrier to recruitment was the requirement for sexual partners to be consented, enrolled, and involved in adverse event (AE) reporting, as many volunteers did not want to disclose their FSAD symptoms to their sexual partners. Therefore, the protocol was amended to allow women to enroll in the trial if they did not have a sexual partner or if they had a sexual partner who did not want to enroll in the study and be involved in AE reporting. In the latter case, consistent with good clinical practice (GCP), investigational product (IP) was only used for un-partnered sexual events to limit un-consented partners’ exposure to IP. If a partnered sexual event was recorded with an un-consented partner, the participant was discontinued from the study. If an un-partnered woman acquired a new sexual partner during the study, she was discontinued, as this life change could impact product efficacy endpoints.

The first screening occurred in July 2021 and the last participant visit was in April 2023. Supplemental Table 1 outlines the schedule of evaluations, previously reported.^10-12^ In brief, volunteers underwent informed consent and screening safety procedures at visit 1 (V1). At all visits, including V1, participants answered the Sexual Function Questionnaire (SFQ28) and the Female Sexual Distress Scale – Desire, Arousal, Orgasm (FSDS-DAO) 1-month recall questionnaires. At visit 2 (V2), participants received an electronic diary (eDiary), which queried participants daily regarding AEs, concomitant medications, and sexual activity. All participants then commenced a 28-day no-drug run-in period after V2, to confirm they would correctly utilize the eDiary. At visit 3 (V3), they commenced a 28-day single-blind placebo run-in period. Data obtained at V4, and eDiary data obtained during the single-blind placebo run-in period were considered baseline data. Enrollment and randomization occurred at V4, and the double-blind dosing period lasted 12 weeks, from V4 to visit 7 (V7).

As previously reported,^10-12^ the co-primary efficacy endpoints were assessed at each monthly visit using validated, 1-month recall questionnaires including (i) the Arousal Sensation (AS) domain (questions 6– 9) of the SFQ28^13^^,^^14^ and (ii) Question 14 (Q14) of the FSDS-DAO. The AS domain of the SFQ28 questionnaire is made up of questions 6, 7, 8, and 9 (Supplemental Table 2). These 4 questions were answered with a Likert scale ranging from 1– 5, with 1 being no sensation and 5 being a very strong sensation. The range of scores for the AS domain was 4– 20, with higher scores indicating higher sexual function. The FSDS-DAO survey included 15 questions, each of which began with the phrase “How often in the past 30 days did you feel” with responses of 0– 4, with 0 indicating never feeling this way and 4 indicating always feeling this way (Supplemental Table 2). Question 14 (Q14) asked “how often in the past 30 days did you feel concerned by difficulties with sexual arousal?”. The range of scores for Q14 of the FSDS-DAO was 0– 4 with lower scores indicating decreased levels of sexual distress.

The eDiary prompted the participant every 24 hours to report whether there had been a sexual event. If there was a sexual event, questions regarding the event had to be completed in the eDiary within 24 hours of the event. The eDiary asked whether the event was partnered or “by myself.” Finally, the eDiary prompted the participant to answer with a Yes or No response “Did you consider sexual activity satisfactory for you?”. The secondary efficacy endpoints of the trial were the change from baseline at week 12 in the mean number and proportion of satisfactory sexual events (SSEs). Finally, exploratory efficacy endpoints included the change from baseline at week 12 in the Desire (questions 1– 4, 14 and 26) and Orgasm (questions 22– 24) domains of the SFQ28. Pre-specified exploratory endpoints are detailed in Supplemental Table S2. At the end of the study, all participants completed a Patient Global Impression of Change Survey and from these data, an independent study group calculated clinically meaningful changes to the primary efficacy endpoints.

### Randomization

Participants were randomized in a 1:1 ratio to either 2-gram Sildenafil Cream or 2-gram Placebo Cream, via blinded and concealed interactive response technology. There were no allocation errors.

### Study product

The placebo cream contained the same ingredients as in the Sildenafil Cream, but without the active ingredient, sildenafil citrate. IP was stored at room temperature (20–25°C) in a secure, temperature and humidity monitored area accessible only to the Investigator or their designee at each study site.

### Investigational product dosing

Each month, IP was dispensed in a 30-gram tube, along with 9, 2-gram dosing cards. Participants were instructed to apply no more than one 2-gram application of product per calendar day and no more than 9 applications per month. Women applied IP ~10– 20 minutes prior to the sexual event. One gram was applied externally to the anterior labial commissure, prepuce of the clitoris, glans, frenulum, vestibule, and labia minora. One gram was applied to the anterior distal vagina to an approximate depth of 0–3 cm or about halfway between the distal and proximal interphalangeal joints, targeting the distal, lower 1/3 of the vagina.

### Subset groups and statistical analysis

Participants in the intent-to-treat (ITT) population, defined as all participants who applied IP at least once during the 12-week double-blind dosing period, were analyzed for these subset analyses. Although the subset analyses based on age and racial group and hormonal contraceptive use were pre-planned, the study was not powered to detect significant differences in these subset analyses and multiple comparisons were not done. All post-hoc analyses reported are exploratory. All subset analyses were performed by an independent data management team, not the study sponsors.

Hormonal contraceptive use included any ethinyl estradiol plus progestin or progestin-only contraceptive method, whether given systemically (eg, oral contraceptive pills) or via local micro-dose formulations (eg, levonorgestrel intrauterine systems). Age group was pre-defined as ≥18 years and ≤ 45 years versus >45 years and no sensitivity analysis was added for other age group categories. Race group was defined as White or non-White. Sexual events were categorized as partnered or un-partnered in the eDiary. After study completion, the medical history and concomitant medication data were reviewed. Daily psychiatric medication users were participants who took one or more psychiatric medications (eg, selective serotonin reuptake inhibitors (SSRIs), selective serotonin norepinephrine reuptake inhibitors (SNRIs)) once daily, versus using psychiatric medications as needed for symptoms (eg, alprazolam for panic attacks, insomnia). Finally, women who per the protocol only used IP during un-partnered sexual events (un-partnered women or women who had sexual partners who did not provide informed consent) were differentiated from women whose sexual partners enrolled in the study and who therefore could use IP during either partnered or un-partnered sexual events.

The SFQ28 domains and FSDS-DAO survey items have previously been reported.^13^^,^^14^ Unless otherwise noted, categorical variables were summarized using counts and percentages. Continuous variables were summarized using the number of observations (n), mean, standard deviation (SD), median, 25^th^ percentile (Q1), 75^th^ percentile (Q3), minimum, and maximum. The efficacy endpoint data were not normally distributed and therefore the assessments between the Placebo Cream and Sildenafil Cream participants based on various subsets were compared using a Wilcoxon-Mann Whitney test with a significant level of *P* < 0.05.

## Results

As shown in Supplemental Fig. S1, volunteers (n = 833) underwent informed consent. Of the consented subjects, 277 eligible participants attended V2 and entered the no-drug run-in period. At V3, 252 participants entered the single-blind placebo run-in period. Two hundred participants were randomized to Sildenafil Cream (n = 101) versus Placebo Cream (n = 99) at V4. The ITT population included 99 Sildenafil and 93 Placebo randomized participants, who received at least one dose of the assigned IP. Table 1 displays the demographics and baseline characteristics of subjects in the ITT population, based on randomization assignment and subset populations of interest. The first screening was in July 2021 and the last participant visit was in April 2023.

**Table 1:** Demographic and baseline characteristics of participants intent to treat population.

Participant subset categorical variables	Participants sildenafil (N = 99)		Participants placebo (N = 93)		*P* value
	N	% Total product group	N	% Total product group	
Age Group					
Age ≥ 18 and ≤ 45 years	88	88.9	82	88.2	.88
Age > 45 years	11	11.1	11	11.8	
Race Categorization for Subset Analyses					
Hispanic or Non-Hispanic Non-White	16	16.2	17	18.3	.70
Hispanic or Non-Hispanic White	83	83.8	76	81.7	
Hormonal Contraceptive Use at Baseline Visit 4					
Yes	56	56.6	46	49.5	.32
No	43	43.4	47	50.5	
Daily Psychiatric Medication Use During the Study					
Yes	33	33.3	30	32.3	.87
No	66	66.7	63	67.7	
Partner Enrollment Status at Baseline					
Un-Partnered	4	4.0	8	8.6	.37
Partner Not Enrolled in Study (Only un-partnered sexual events using study product allowed)	4	4.0	2	2.2	
Partner Enrolled in Study (Un-partnered and partnered sexual events using study product allowed)	91	91.9	83	89.2	

Based on responses to the Patient Global Impression of Change survey, from the intent to treat population, we found that a clinically meaningful change for the SFQ28 arousal sensation domain was 1.5 ± 3.26 from baseline to end-of-study and for the FSDS-DAO question 14, we found that a mean change of −0.5 represented a clinically meaningful change from baseline.

Sildenafil Cream users reported more sexual events in the eDiary during the double-blind dosing period (n = 1357), but this was not statistically different than the 1160 sexual events reported in the eDiary by Placebo Cream users (*P* = .56). Sildenafil Cream users reported significantly more un-partnered sexual events (23.1% of total Sildenafil exposed sexual events) compared to Placebo Cream users (19.7% of total Placebo exposed sexual events) (*P* < 0.01) during the double-blind dosing period.

Age group, race group, and baseline hormonal contraceptive use did not significantly affect the co-primary endpoints of the SFQ28 AS domain and FSDS-DAO Q14 (Table 2). Although not statistically significant, non-White women assigned to Sildenafil Cream had a mean increase in their SFQ28 AS domain score of 3.3 ± 1.09 from baseline at week 12, compared to non-White women assigned to Placebo Cream, who had a mean increase of 1.14 ± 2.5 (*P* = .12). Table 2 also demonstrates that among Sildenafil Cream assigned users, participants aged 18– 45 years, non-White participants, and hormonal contraceptive users had larger improvements from baseline in the co-primary endpoint of the SFQ28 AS domain, compared to Sildenafil Cream users who were > 45 years old, White, or who did not use hormonal contraceptives (formal statistical comparisons of within treatment groups were not part of the pre-planned subset analyses).

**Table 2:** Subset population results based on product use and visit.

Variable and Subset Population	Sildenafil		Placebo		Sildenafil – Placebo		*P* value
	Least squares (LS) mean change from baseline	Standard error (SE) in LS mean change from baseline	Least squares (LS) mean change from baseline	Standard error (SE) in LS mean change from baseline	Difference in LS mean (Sildenafil – Placebo)	SE in difference of LS mean	
Change from Baseline to Week 12, Primary Endpoint, SFQ28 AS Domain							
Age Group ≥18 years and ≤ 45 years	1.2	0.42	0.8	0.46	0.4	0.62	.51
Age Group >45 years	−0.6	1.23	0.5	1.14	−1.1	1.68	.52
Race = White	0.7	0.40	0.7	0.44	-0.1	0.60	.91
Race = Non-White	3.3	1.09	0.8	1.14	2.5	1.59	.12
Baseline Hormonal Contraceptive Use: Yes	1.7	0.58	0.7	0.59	1.0	0.83	.22
Baseline Hormonal Contraceptive Use: No	0.7	0.53	0.8	0.59	−0.1	0.80	.90
Change from Baseline to Week 12, Primary Endpoint, FSDS-DAO Question 14							
Age Group ≥18 years and ≤ 45 years	−0.2	0.12	−0.2	0.13	−0.1	0.17	.74
Age Group >45 years	−0.7	0.47	−0.6	0.44	−0.1	0.65	.89
Race = White	−0.2	0.12	−0.2	0.13	0	0.17	.88
Race = Non-White	−0.5	0.38	−0.5	0.37	0	0.53	.93
Baseline Hormonal Contraceptive Use: Yes	−0.3	0.16	0.1	0.17	−0.4	0.23	.11
Baseline Hormonal Contraceptive Use: No	−0.2	0.16	−0.4	0.17	0.2	0.23	.35
Change from Baseline to Week 12, Secondary Endpoint, Mean SSEs							
Age Group ≥18 years and ≤ 45 years	0.1	0.26	0	0.28	0.1	0.38	.73
Age Group >45 years	−0.5	0.67	-0.1	0.66	−0.4	0.95	.67
Race = White	0.0	0.27	0.3	0.30	−0.3	0.40	.45
Race = Non-White	0.7	0.63	−1.5	0.58	2.3	0.86	.02
Baseline Hormonal Contraceptive Use: Yes	−0.4	0.34	−0.3	0.37	0	0.50	.94
Baseline Hormonal Contraceptive Use: No	0.4	0.35	0.1	0.38	0.2	0.51	.68
Un-Partnered Sexual Events	−0.12	0.27	−0.04	0.32	−0.08	0.42	.85
Partnered Sexual Events	0.07	0.27	−0.01	0.29	0.08	0.40	.85
Change from Baseline to Week 12, Exploratory Endpoint, SFQ28 Desire Domain							
Daily Psychiatric Medication Use: Yes	−1.08	0.78	−1.98	0.90	0.90	1.19	.45
Daily Psychiatric Medication Use: No	1.85	0.55	0.52	0.58	1.33	0.80	.10
Change from Baseline to Week 12, Exploratory Endpoint, SFQ28 Orgasm Domain							
Un-Partnered Women or Sexual Partner Not Enrolled (Only un-partnered sexual events using study product allowed)	2.39	0.95	−0.19	0.75	2.58	1.27	.06
Women with Enrolled Sexual Partners (Un-partnered and partnered sexual events using study product allowed)	0.42	0.34	0.67	0.37	−0.24	0.50	.63

Age group and baseline hormonal contraceptive use also did not impact the change in the mean number of SSEs from baseline to week 12 (Table 2, all *P* values >0.67). Non-White women randomized to Sildenafil Cream recorded an increase of 0.7 ± 0.63 SSEs at week 12 while non-white Placebo Cream users recorded a decrease in SSEs (−1.5 ± 0.58) at week 12 (*P* = .02).

The change from baseline in SFQ28 Desire domain scores decreased at week 12 among daily psychiatric medication users, whether randomized to Sildenafil Cream (−1.08 ± 0.78) or Placebo Cream (−1.98 ± 0.90) (*P* = .45) (Table 2). Among non-users of daily psychiatric medications, those receiving Sildenafil Cream reported an increase in their SFQ28 Desire domain scores (1.85 ± 0.55) which was more than 3 times higher than the increase reported by Placebo Cream users (0.52 ± 0.58), but this was not statistically significant (*P* = .10), likely due to the smaller subset sample size (Table 2).

Non-White participants were significantly more likely than White participants to be un-partnered (18.4% versus 6.1%, respectively) (*P* < .01) or to have partners who did not want to enroll in the study (7.9% versus 4.6%, respectively) (*P* = .02). These trends were also significant for non-Hispanic Black women (29.4% were un-partnered and 11.8% had un-enrolled partners) (*P* < .01 versus White women). The proportion of un-partnered women and those with un-enrolled partners was similar among Hispanic versus non-Hispanic women (*P* = .70). Among this small subset of women who only used IP during un-partnered sexual events, Sildenafil Cream users (n = 8, Table 1) reported a 2.39 ± 0.95 increase in their SFQ28 Orgasm domain scores at week 12, while Placebo Cream users (n = 10, Table 1) reported a decrease (−0.19 ± 0.75) (*P* = .06) (Table 2).

## Discussion

These subset analyses are relevant to demographic, behavioral, and medication use factors which could potentially impact the efficacy of FSAD treatment. Although the frequency of sexual activity has been related to age,^15^ we found that age group did not significantly affect either co-primary efficacy endpoint. Within the Sildenafil Cream group, we observed that participants aged 18– 45 years had greater improvement (1.2 ± 0.42) from baseline in the co-primary endpoint of the SFQ28 AS domain, compared to participants >45 years old (−0.6 ± 1.23). Additionally, we found that race did not significantly affect either co-primary efficacy endpoint. We noted that within the Sildenafil Cream group, non-White participants had greater improvement (3.3 ± 1.09) from baseline in the co-primary endpoint of the SFQ28 AS domain, compared to White participants (0.7 ± 0.40).

Effective contraceptive use is standard GCP during studies of investigational products. Hormonal contraception was used by approximately half of study participants, who had an average age of 36 years, consistent with US national data on contraceptive use.^16^^,^^17^ Hormonal contraceptive use has been associated with lower sexual desire as well as reduced sexual arousal,^18-20^ but also has many non-contraceptive health benefits.^21^ We did not find that hormonal contraceptive use had any significant impact on the primary or secondary efficacy endpoints. Within the Sildenafil Cream group, we noted that hormonal contraceptive users had greater improvement (1.7 versus 0.7) from baseline in the co-primary endpoint of the SFQ28 AS domain compared to women who did not use hormonal contraceptives. We were not able to separate out systemic hormonal contraceptive use versus micro-dose hormonal contraceptive exposure (eg, levonorgestrel intrauterine system) or ethinyl estradiol-containing versus progestin-only contraceptives, and this is a limitation of the analyses.

Our findings of lower SFQ28 Desire domain scores among daily psychiatric medication users, whether assigned to Sildenafil Cream or Placebo Cream, are consistent with this well-known side effect of these medications.^22-24^ Approximately one third of study participants used at least one daily psychiatric medication, most commonly Norepinephrine-dopamine reuptake inhibitors (NDRI), SSRIs, or SNRIs, which is consistent with national data.^25^

Among the ITT population, Sildenafil Cream users reported more sexual events than Placebo Cream users and had significantly more un-partnered sexual events, but this did not translate into higher mean numbers of SSEs for these un-partnered sexual events. The majority of women in the ITT population had both partnered and un-partnered sexual events and therefore we could not correlate individual un-partnered sexual events during the month with the monthly recall assessments at the follow up visits.

The subset of women who, by protocol rules, could only use IP during solo sexual events were un-partnered women or women with un-enrolled sexual partners. This small subset reported higher SFQ28 Orgasm domain scores with Sildenafil Cream use compared to Placebo use (*P* = .06). While this is consistent with past data supporting more intense and frequent orgasms with masturbation,^26-28^ it is reassuring that Sildenafil Cream use increased the Orgasm endpoint during solo sexual events over that measured with Placebo Cream use. We recognize that this small subset had other differences compared to the larger ITT population, including that they were more likely to be Hispanic or non-Hispanic non-White. For women with un-enrolled sexual partners, we do not know if the sexual partner knew that the woman was enrolled in the trial and/or the indication for the study product. There was a signal in this study that non-White women randomized to Sildenafil Cream had significantly more SSEs, but this subset was very small, and given our data on the race and ethnicity of un-partnered women and sexual partner enrollment, we suspect that this comparison is likely impacted by additional factors rather than race and ethnicity alone.

Through pre-planned subset analyses, this study explored factors (demographic, behavioral, and medication use) which could potentially impact the efficacy of FSAD treatment. Limitations of this study, in addition to those noted above, include that the study population consisted mostly of Hispanic and non-Hispanic White women, partnered women, and young women, although this is similar to previous treatment studies for hypoactive sexual desire disorder.^29-34^ Any subset analysis will obviously have reduced sample cell sizes and thus statistical comparisons are limited and type I and II errors may occur.

There are currently no US FDA approved treatments for FSAD, and therefore studies such as this are important to inform efficacy evaluations of potential treatments. Although this large study had strict inclusion and exclusion criteria, we believe these subset analyses addressed important variables which could impact treatment efficacy and will help us best tailor future registration trials.

## Supplementary Material

20240801_Supp_Figure_1_Participant_Dispo_Subset_qfae079

Supplemental_qfae079

## Clinical Trial Registration

ClinicalTrials.gov NCT04948151.
