# Supplementary figures and images for: Impact of age, race, and medication use on efficacy endpoints in a randomized controlled trial of topical sildenafil cream for the treatment of female sexual arousal disorder
Source: Sex Med. 2024 Nov 19;12(5):qfae079. doi: 10.1093/sexmed/qfae079 (PMC11576099; doi:10.1093/sexmed/qfae079)

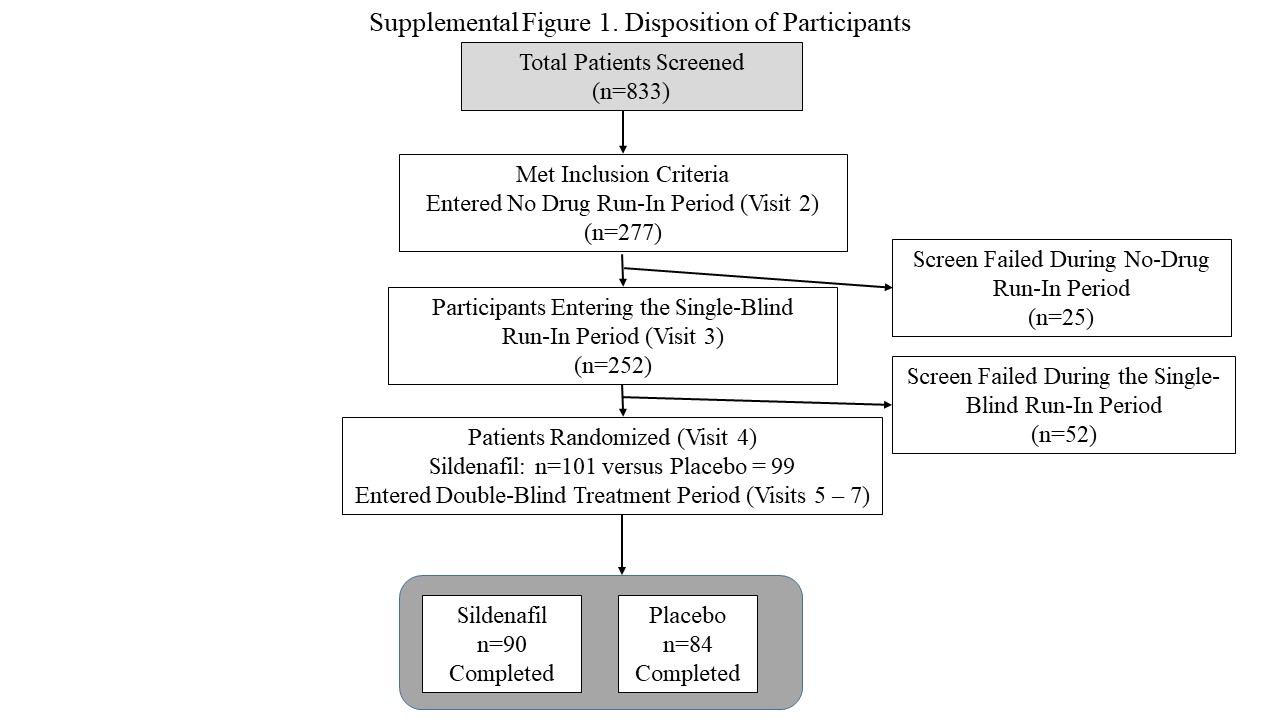

Supplement: 20240801_Supp_Figure_1_Participant_Dispo_Subset_qfae079 [file 20240801_supp_figure_1_participant_dispo_subset_qfae079.jpeg]
