# Supplementary material for: Impact of age, race, and medication use on efficacy endpoints in a randomized controlled trial of topical sildenafil cream for the treatment of female sexual arousal disorder
Source: Sex Med. 2024 Nov 19;12(5):qfae079. doi: 10.1093/sexmed/qfae079 (PMC11576099; doi:10.1093/sexmed/qfae079)
Supplement: Supplemental_qfae079 [file supplemental_qfae079.docx]

**Figure Legend:**

**Supplemental Figure 1.** Disposition of Participants

**Supplemental Table 1.** Schedule of Eligibility Events and Efficacy Assessments

| Assessment Category | Assessments | Medical Screening | Start of No Drug Run-In | Start of Single-Blind Placebo Run-In | Start of Each Month of Double-Blind Dosing Period | | | | Follow-Up Assessment |
| --- | --- | --- | --- | --- | --- | --- | --- | --- | --- |
|  | Visit | 1 | 2 | 3 | 4 | 5 | 6 | 7 | Phone Call |
|  | Study Weeks | 0 | 1 | 5 | 9 | 13 | 17 | 21 | 22 |
|  | Study Days | Day -28 to -1 | 0 | Visit 2 + 28 -31 days | Visit 3 + 28 – 31 days | Visit 4 + 28 – 31 days | Visit 5 + 28 – 31 days | Visit 6 + 28 – 31 days | Visit 7 + 4 – 10 days |
| Informed Consent^a^ | Informed Consent | X |  |  |  |  |  |  |  |
|  | Inclusion/Exclusion | X^a^ | X^a^ | X | X | X | X | X |  |
|  | Medical History | X^a^ |  |  |  |  |  |  |  |
| Eligibility Determination | Clinical Interview | X |  |  |  |  |  |  |  |
|  | PHQ-8 | X | X | X | X |  |  |  |  |
|  | GAD-7 | X | X | X | X |  |  |  |  |
| Study Administration | Randomization |  |  |  | X |  |  |  |  |
| Efficacy Assessments | FSDS-DAO | X |  | X^b^ | X | X | X | X |  |
|  | SFQ28 | X |  | X^b^ | X | X | X | X |  |
|  | eDiary |  | X | X^b^ | X | X | X | X |  |

a. For both Patient and her Partner (all partners are required to attend Visits 1 and 2; same sex partners of childbearing potential will also attend Visit 7). Partners are permitted to perform vital signs utilizing a study-allocated blood pressure cuff from home. If self-assessment is chosen, partners will be seen remotely through a telemedicine visit.

b. Responses and scores following the 4-week single blind placebo run-in will be utilized to establish baseline.

**Supplemental Table 2. Primary, Secondary and Exploratory Efficacy Endpoints Assessed in the Sildenafil Cream 3.6% Study^10^**

**Appendix 2. Efficacy Endpoints**

| Source | Endpoint Domain Name | Questions | Question Text | Answer Scale and Text | Domain Range | Score Suggesting Normal Function |
| --- | --- | --- | --- | --- | --- | --- |
| SFQ28 | Arousal Sensation | 6 | Over the last 4 weeks, how often did you have a feeling of ‘warmth’ in your vagina/genital area when you took part in sexual activity? | (NA) I did not take part in sexual activity (1) Not at all (2) Sometimes (3) Often (4) Very Often (5) Every time | 4 - 20 | ≥ 14 |
|  |  | 7 | Over the last 4 weeks in general, how much ‘warmth’ did you feel in your vagina/genital area when you took part in sexual activity? | (NA) I did not take part in sexual activity (1) None (2) Slightly ‘warm’ (3) Moderately ‘warm’ (4) Very ‘warm’ (5) Extremely ‘warm’ |  |  |
|  |  | 8 | Over the last 4 weeks, how often did you have a sensation of ‘pulsating’ (‘tingling’) in your vagina/genital area when you took part in sexual activity? | (NA) I did not take part in sexual activity (1) Not at all (2) Sometimes (3) Often (4) Very Often (5) Every time |  |  |
|  |  | 9 | Over the last 4 weeks, in general, how much ‘pulsating’ (‘tingling’) in your vagina/genital area did you notice when you took part in sexual activity? | (NA) I did not take part in sexual activity (1) No sensation (2) A mild sensation (3) A moderate sensation (4) A strong sensation (5) A very strong sensation |  |  |
| SFQ28 | Desire | 1 | Over the last 4 weeks, how often have you had pleasurable thoughts and feelings about sexual activity? | (1) Not at all (2) Rarely (3) Sometimes (4) Often (5) Very Often | 5 - 31 | ≥ 23 |
|  |  | 2 | Over the last 4 weeks, how often have you wanted to be sensually touched and caressed by your partner? | (1) Not at all (2) Rarely (3) Sometimes (4) Often (5) Very Often |  |  |
|  |  | 3 | Over the last 4 weeks, how often have you wanted to take part in sexual activity? | (1) Not at all (2) Rarely (3) Sometimes (4) Often (5) Very Often |  |  |
|  |  | 4 | Over the last 4 weeks, how often have you initiated sexual activity with your partner? | (1) Not at all (2) Rarely (3) Sometimes (4) Often (5) Very Often |  |  |
|  |  | 14 | Over the last 4 weeks, how often did you take part in sexual activity with penetration (e.g., vaginal penetration and intercourse)? | (0) I did not take part in sexual activity (1) Once/twice (2) 3-4 times (3) 5-8 times (4) 9-12 times (5) 13-16 times (6) >16 times |  |  |
|  |  | 26 | Thinking about your sexual life over the last 4 weeks, how often did you look forward to sexual activity? | (1) Not at all (2) Rarely (3) Sometimes (4) Often (5) Very Often |  |  |
| SFQ28 | Orgasm | 22 | Over the last 4 weeks, how often did you have an orgasm when you took part in sexual activity (may be with or without a partner)? | (NA) I did not take part in sexual activity (1) Not at all (2) Sometimes (3) Often (4) Very Often (5) Every time | 1 - 15 | ≥ 12 |
|  |  | 23 | Over the last 4 weeks, in general, how pleasurable were the orgasms that you had? | (0) I did not have any orgasms (1) Not pleasurable (2) Slightly pleasurable (3) Moderately pleasurable (4) Very pleasurable (5) Extremely pleasurable |  |  |
|  |  | 24 | Over the last 4 weeks, in general, how easy was it for you to reach orgasm? | (0) I did not have any orgasms (1) Very difficult (2) Quite difficult (3) Neither easy nor difficult (4) Quite easy (5) Very easy |  |  |
| FSDS-DAO | Total Score | 1 | Distressed about your sex life | All questions start with: Below is a list of feelings and problems that women sometimes have concerning their sexuality. Please read each item carefully and circle the number that describes “How often that problem has bothered you or caused you distress during the past 30 days including today.”  Answers are: (0) Never (1) Rarely (2) Occasionally (3) Frequently (4) Always | 0 - 60 | < 18 |
|  |  | 2 | Unhappy about your sexual relationship |  |  |  |
|  |  | 3 | Guilty about sexual difficulties |  |  |  |
|  |  | 4 | Frustrated by your sexual problems |  |  |  |
|  |  | 5 | Stressed about sex |  |  |  |
|  |  | 6 | Inferior because of sexual problems |  |  |  |
|  |  | 7 | Worried about sex |  |  |  |
|  |  | 8 | Sexually inadequate |  |  |  |
|  |  | 9 | Regrets about your sexual functioning |  |  |  |
|  |  | 10 | Embarrassed about sexual problems |  |  |  |
|  |  | 11 | Dissatisfied with your sex life |  |  |  |
|  |  | 12 | Angry about your sex life |  |  |  |
|  |  | 13 | Bothered by low sexual desire |  |  |  |
|  |  | 14 | Concerned by difficulties with sexual arousal |  |  |  |
|  |  | 15 | Frustrated by problems with orgasm |  |  |  |
